# Chemotherapy for malignant pleural mesothelioma: past results and recent developments

**DOI:** 10.1038/sj.bjc.6600673

**Published:** 2003-01-28

**Authors:** S Tomek, S Emri, K Krejcy, C Manegold

**Affiliations:** 1 Department of Medicine I, Clinical Division of Oncology, Vienna, Austria; 2 Department of Chest Disease, Hacettepe University School of Medicine, Ankara, Turkey; 3 Eli Lilly Regional Operations GesmbH Area Medical Centre, Vienna, Austria; 4 Clinical Division of Oncology, Department of Medicine, Thoraxklinik Heidelberg, Germany

**Keywords:** malignant pleural mesothelioma, chemotherapy

## Abstract

This review summarises the results of previously conducted clinical trials, and subsequently presents data arising from all phase II–III studies on chemotherapy of malignant pleural mesothelioma (MPM) published since the last relevant overview. While response rates exceeding 30% have barely been achieved with established cytotoxic drugs in MPM therapy, novel chemotherapeutic agents and their combinations appear more promising. This applies especially to the antimetabolites, and in particular to pemetrexed that produced response rates of up to 45% in combination with platinum compounds. Raltitrexed combined with oxaliplatin has also been shown to be effective, and gemcitabine–applied as a single agent or in combination with cisplatin–as well as vinorelbine appear to improve the quality of life in patients presenting with MPM. Data can now be more precisely analysed by increasingly implemented randomised studies, applying a standardised staging system, and distinguishing prognostic groups. While chemotherapy for MPM remains a challenging task, important steps have clearly been made in the past years to combat this aggressive disease. The publication of pemetrexed with cisplatin phase III results in a peer-reviewed journal may soon establish a standard of care.

Although MPM aetiology is well known, therapeutic success with this disease has been unsatisfactory. Standard malignant pleural mesothelioma (MPM) therapy is still deficient, and decisions for surgical, radiotherapy or multimodal procedures are made on a case-by-case basis. In the majority of cases, a palliative treatment approach remains the only choice. The possibilities to resect MPM curatively are rare and only given in early-stage disease. Yet even at this point, resection is a matter of dispute because of historically high morbidity and mortality rates, relapse tendencies and disappointing long-term survival rates (Rusch and Venkatraman, 1996; Boutin *et al*, 1998). Irradiation for MPM assists in repelling tumour growth and temporarily relieving pain, but not attaining appreciably lengthened overall survival time (Kutcher *et al*, 1987; Soubra *et al*, 1990; Bissett *et al*, 1991). There is also no clear evidence that multimodality protocols involving surgery, radiotherapy and chemotherapy can improve survival (Antman *et al*, 1980; Hilaris *et al*, 1984; Linden *et al*, 1996). Although success in MPM chemotherapy has been limited, it now gives rise to promise by the use of an established and standardised staging system, with consideration given to prognostic factors and development of novel cytostatic agents.

## ROLE OF CHEMOTHERAPY

Chemotherapy for MPM continues to be challenging. A multitude of cytotoxic drugs have been tested both as single agents and in combination chemotherapy regimens. The rates of objective tumour regression have only been between 10 and 30% with cytotoxic monotherapy, having no significant impact on median survival. Combination chemotherapy has shown no clear advantage over single-agent therapy (Linden *et al*, 1996).

The evaluation of systemic chemotherapy in MPM has been problematic for several reasons. Owing to the rarity of this disease, only a few randomised studies based on large numbers of participants have been implemented to provide statistically significant statements regarding response to a particular therapy.

Furthermore, inadequate imaging procedures and nonuniform staging systems have complicated data interpretation. As performed in early studies, restaging by means of chest radiography failed to determine response to a given therapy accurately. Response rates have been more reliable since CT scanning has been recognised as a diagnostic tool. In 1995, the International Mesothelioma Interest Group (IMIG) proposed a novel TNM staging system, which was designed to record data concerning the natural history of the disease and was validated in two large surgical series of mesothelioma patients (Rusch and Venkatraman, 1996; Pass *et al*, 1998). The universal application of this system has ever since allowed for a more prudent evaluation of results emerging from clinical studies (Pass *et al*, 1998; Steele and Rudd, 2000).

Early clinical trials of MPM patients included heterogeneous groups of patients with divergent risk factors and were therefore often not powerful enough in assessing therapeutic efficacy of a particular treatment. In 1998, the European Organization for Research and Treatment of Cancer (EORTC) identified several prognostic variables for the course of the disease. In a multivariate analysis of the EORTC, poor prognosis was associated with the sarcomatous histologic subtype, male gender, poor performance status and a high white blood cell count (Curran *et al*, 1998). Likewise, the Cancer and Leukemia Group B (CALGB) analysed several pretreatment factors pooled from seven phase II studies that were predictive of poor survival and defined six prognostic groups. Poor prognosis was seen in patients with the following criteria: age older than 75 years, poor performance status, chest pain, dyspnoea, weight loss, high white blood cell count, elevated platelet count, low haemoglobin, elevated serum lactate dehydrogenase levels, pleural effusion and nonepithelial histology.

Owing to the establishment of prognostic scoring systems, identification of risk groups facilitated improved study design by evaluating more homogeneous patient groups or risk-group stratification in the course of randomisation (Curran *et al*, 1998).

The following sections summarise conventional single-agent and combination chemotherapy strategies. Results emerging from recent clinical studies with novel cytotoxic agents and their combinations are then presented, which have been published since the last review of chemotherapy in MPM.

Tables 1

**Table 1:** Series of ⩾15 patients with MPM treated with single-agent chemotherapy since 1995

**Single agent**	**First author (year)**	**No. of patients**	**Responders**	**95% confidence interval (%)**	**Median survival (months)**	
			***No***	**%**		
**Anthracyclines and related compounds**						
Liposomal doxorubicin	Baas (2000)	32	2	6	0–20	13
Liposomal doxorubicin	Oh (2000)	24	0	0	na	9.3
Liposomal danaurubicin	Steele (1998)	11	0	0	na	6.1
						
**Platinum compounds**						
ZD0473	Giaccone (2001)	10	Two regressions of evaluable disease	na	na	
						
**Alkylating agents**						
Ifosfamide	Andersen (1999)	26	1	4	0–11	10
Etoposide i.v.	Sahmoud (1997)	49	2	4	1–15	7.3
Etoposide p.o.	Sahmoud (1997)	45	3	7	2–20	9.5
**Topoisomerase interactive agents**						
*Campthotecin analogues*						
Irinotecan	Kindler (2000)	28	0	0	10–55	7.9
Topotecan						
	Maksymiuk (1998)	22	0	0	na	8
**Antimicrotubule agents**						
*Vinca alcaloids*						
Vinorelbine	Steele (2000)	64	12	21	10–44	13.4
Vincristine	Martensson (1989)	23	0	0	0–14	7
Vinblastine	Cowan (1988)	20	0	0	0–16	3
*Taxanes*						
Docetaxel	Belani (1999)	19	1	5	0–26	na
Docetaxel	Vorobiof (2000)	22	3	14	7–46	12
Paclitaxel	v. Meerbeck (1996)	25	0	0	0–15	9.8
Paclitaxel	Vogelzang (1999)	35	Three regressions of evaluable disease	2–10	5	
						
**Antimetabolites**						
Edatrexate	Kindler (1999)	20	5	25	9–49	9.6
Edatrexate+LV-rescue	Kindler (1999)	38	6	16	6–31	6.6
Gemcitabine	Kindler (2001)	17	0	0	3–13	4.1
Gemcitabine	van Meerbeck (1999)	27	2	7	1–24	8
Gemcitabine	Bischoff (1998)	16	5	31	na	na
Pemetrexed	Scagliotti (2001)	62	9	6	na	10.7

na=not applicable.

2

**Table 2:** Series of ⩾15 patients with MPM treated with combination chemotherapy since 1995

**Agent**	**First author (year)**	**No. of patients**	**Responders**	**95% confidence interval (%)**	**Median survival**	
			No.	%		
**Doxorubicin-containing combinations**						
Doxorubicin+cisplatin+mitomycin	Pennucci (1997)	23	5	21	7 – 42	11
Doxorubicin+cisplatin+mitomycin+bleomycin	Breau (1991)	25	11	44	27 – 63	na
						
**Cisplatin-containing combinations**						
Cisplatin+DHAC	Samuels (1998)	29	5	17	5 – 30	6.4
Cisplatin+etoposide	Eisenhauer (1988)	26	3	12	4 – 30	na
Cisplatin+gemcitabine	Byrne (1999)	21	10	48	26 – 69	10.3
Cisplatin+gemcitabine	Novak (2002)	52	17	33	20 – 46	11.2
Cisplatin+gemcitabine	Van Haarst (2000)	22	4	15	na	10
Cisplatin+Irinotecan	Nakano (1999)	15	4	27	8 – 55	7.1
Cisplatin+mitomycin+vinblastine	Middleton (1998)	39	8	20	na	6
Cisplatin+pemetrexed (phase I)	Thodtman (1999)	11	5	45	na	na
Cisplatin+paclitaxel	Fizazi (2000)	18	1	6	0 – 24	12
**Other combinations**						
Methotrexate+mitoxantrone+mitomycin	Pinto (2001)	22	6	32	12 – 51	13.5
Carboplatin+gemcitabine	Aversa (1998)	18	3	16	na	8.6
Carboplatin+pemetrexed (phase I)	Hughes (2002)	25	8	32	na	15
Oxaliplatin+raltitrexed	Fizazi (2000)	30	9	30	15 – 49	na
Oxaliplatin+vinorelbine	Steele (2000)	17	2	12	na	na
Docetaxel+irinotecan	Knuuttila (2000)	15	0	0	2 – 45	8.5

na=not applicable.

and 3

**Table 3:** Series of randomized phase II – III studies in patients with MPM treated with chemotherapy

**Agent**	**First author (year)**	**No. of patients**	**Responders**	**95% confidence interval (%)**	**Median survival (months)**	
			**No.**	**%**		
Doxorubicin *vs* cyclophosphamide	Sorensen (1985)	32	0	0	0–19	na
			0	0	0–19	
Doxorubicin+cyclophosphamide *vs* doxorubicin+cyclophosphamide+DTIC	Samson (1987)	76	4	11	6–21	7.5
			5	13	6–21	6.3
Cisplatin+doxorubicin *vs* cisplatin+mitomycin	Chahinian (1993)	70	5	14	5–30	
			5	26	12–43	7.7
						
Cisplatin+etoposide *vs* carboplatin	White SC (2000)	25	1	8	na	4.7
			0	0		5.4
						
Onconase *vs* doxorubicin	Vogelzang (2000)	154	na	na	na	7.7
						8.2
Cisplatin *vs* cisplatin+pemetrexed	Vogelzang (2002)	456	na*	na*	na*	na*
**Ongoing trials agent**	**No. of patients planned**					
Doxorubicin *vs* doxorubicin+onconase	300					
Cisplatin *vs* cisplatin+raltitrexed	240					
Pemetrexed *vs* best supportive care	240					
Vinorelbine *vs* MVP *vs* best supportive care	840					
Cisplatin+gemcitabine *vs* cisplatin+gemcitabine+bevacizumab	106					

* Vogelzang N.: http://www.asco.org/asco/ascoMainConstructor/1,47468,_12|002351,00.asp?cat=General+Oncology.

na=not applicable.

summarise phase II–III single-agent and combination chemotherapy trials for MPM including more than 15 patients, which have been conducted since 1995.

### Single-agent chemotherapy

Doxorubicin is the most frequently investigated chemotherapeutic agent in the treatment of MPM. Studies have failed to corroborate evidence, however, for encouraging response rates to doxorubicin of up to 20% that had been reported in earlier studies (Aisner and Wiernik, 1981; Antman and Corson, 1985). Likewise, newer anthracyclines such as epirubicin, detorubicin, pirarubicin and mitoxantrone have shown low levels of efficacy and have offered no clinically relevant advantage over doxorubicin (Colbert *et al*, 1985; Eisenhauer *et al*, 1986; Sridhar *et al*, 1989; Kaukel *et al*, 1990; Magri *et al*, 1991; van Breukelen *et al*, 1991; Mattson *et al*, 1992; Magri *et al*, 1992). In summary, the overall response rate produced by anthracyclines applied in MPM appears to be no higher than 15% and median survival does not exceed 8 months.

Apart from anthracyclines, several studies have investigated the platinum compounds, cisplatin and carboplatin. Single-agent cisplatin resulted in a response rate of merely 14.3% and a median survival of 7.5 months (Zidar *et al*, 1988). Studies using the newer compound carboplatin resulted in similar response rates ranging between 6 and 16% (Mbidde *et al*, 1986; Raghavan *et al*, 1990; Vogelzang *et al*, 1990).

The alkylating agents cyclophosphamide and mitomycin have shown low-level activity in MPM therapy (Bajorin *et al*, 1987; Sorensen *et al*, 1985). Promising results initially arising from high-dose ifosfamide therapy (Alberts *et al*, 1988) could not be confirmed by subsequent studies (Falkson *et al*, 1992; Zidar *et al*, 1992; Icli *et al*, 1996; Krarup-Hansen, 1996; Andersen *et al*, 1999).

The older vinca alkaloids vinblastine, vincristine and vindesine have demonstrated no activity in the treatment of MPM (Kelsen *et al*, 1983; Boutin *et al*, 1987; Cowan *et al*, 1988; Martensson & Sorenson, 1989). Likewise, poor results were shown with oral as well as IV etoposide (Tammilehto *et al*, 1994; Sahmoud *et al*, 1997).

The antifolates methotrexate and edatrexate have been the only single agents to produce comparatively better results. A Norwegian study reported a satisfactory response rate of 37% for high-dose methotrexate and a median survival of 11 months, for trial subjects with an epithelial subtype drawing a particular benefit from this chemotherapy option (Solheim *et al*, 1992). Sequential multicentre phase II studies conducted by the CALGB have evaluated the activity of the folate antagonist edatrexate, with and without leucovorin rescue. Edatrexate produced 25% overall response rates but proved to be relatively toxic. Leucovorin rescue in the control arm led to decreased toxicities, but may also have reduced the agent's efficacy (Kindler *et al*, 1999). Other antimetabolites like fluorouracil, dihydro-5-azacytidine (DHAC), dideazafolic acid and trimetrexate have shown minor or no activity in the treatment of MPM (Harvey *et al*, 1984; Vogelzang *et al*, 1994; Vogelzang *et al*, 1997; Samuels *et al*, 1998).

### Combination chemotherapy

#### Doxorubicin-based regimens

Doxorubicin is the anthracycline most frequently included in chemotherapeutic regimens. Initially encouraging response rates experienced with the doxorubicin–cisplatin combination in two phase II studies carried out in Germany (RR: 46%) and Italy (RR: 25%) (Henss *et al*, 1988; Ardizzoni *et al*, 1991) failed to be confirmed by a subsequent, randomised CALGB study of doxorubicin–cisplatin *vs* mitomycin–cisplatin treatment (Chahinian *et al*, 1993). The doxorubicin-cisplatin doublet was only able to produce a 14% response rate and proved inferior to the mitomycin–cisplatin combination (RR: 26%). Median survival duration from study entry was 7.7 and 8.8 months, respectively, with no significant differences between treatments. An Italian group administered the triplet doxorubicin–cisplatin–mitomycin to 24 MPM patients and reported a response rate of 20.9%. Thus, the observed level of activity was similar to that obtained with the respective doublets (Pennucci *et al*, 1997).

The combination of doxorubicin, cisplatin and cyclophosphamide was tested by another prospective trial and produced a similar rate of response (Shin *et al*, 1995). A multi–institutional randomised study was designed to compare the activity of doxorubicin-cyclophosphamide with a triplet consisting of these agents in addition to dacarbazine. With a response rate of 13%, the triplet did not prove superior to the doublet (RR: 11%) (Samson *et al*, 1987).

The combination of doxorubicin and ifosfamide has also been investigated within two studies. One study giving doxorubicin and ifosfamide every 3 weeks achieved a response rate of 12.5% (Carmichael *et al*, 1989). Based on the same trial design, another group applied dose-escalated doxorubicin at 75 mg m^−2^, with a 32% response rate (Dirix *et al*, 1994). However, the 7-month median survival was poor and toxicity high, limiting the value of this schedule in the treatment of MPM.

Disappointing response rates have also been shown by the anthracyclines epirubicin and rubidazone as combined with ifosfamide and dacarbazine (Zidar *et al*, 1983; Magri *et al*, 1992).

#### Cisplatin-based regimens

Apart from combinations with anthracyclines, cisplatin has also been tested in numerous other chemotherapy regimens. Three trials evaluated cisplatin combined with etoposide, showing response rates from 12 to 24% (Eisenhauer *et al*, 1988; Planting *et al*, 1995; White *et al*, 2000). The doublet combination of cisplatin and DHAC attained an insufficient response rate of 17.3% (Samuels *et al*, 1998). Moderate antitumour activity (RR: 25%) was achieved by the doublet cisplatin–mitomycin in one arm of CALGB 8435, but median survival was poor (7.7 months) (Chahinian *et al*, 1993). A 25% response rate and a 13-month mean duration of response have been indicated for the cisplatin–vinblastine combination (Tsavaris *et al*, 1994). The cisplatin–mitomycin-C–vinblastine triplet did not prove to be superior to the doublets (RR: 23%), yet produced a symptomatic benefit for 63% of the patients with particularly good response for pain (Middleton *et al*, 1998).

Likewise, other treatments combining such conventional cytotoxics as methotrexate with vincristine and mitomycin with vindesine have failed to prove effective (Dimitrov *et al*, 1982; Gridelli *et al*, 1992).

## NOVEL CYTOTOXICS

### Anthracyclines

*Liposomal anthracyclines*

*Liposomal doxorubicin*. Within a phase II study, the EORTC treated 35 patients with liposomal doxorubicin (Caelyx®) at a dosage of 45 mg m^−2^ every 4 weeks (Baas *et al*, 2000). The drug was well tolerated, but the results were disappointing. Only two of the 31 patients (6%) showed a partial response and the median survival was 13 months.

Similar results were reported by Oh *et al*, who administered 50 mg m^−2^ of liposomal doxorubicin (Doxil®) every 4 weeks to 24 patients with pleural mesothelioma (Oh *et al*, 2000). With no objective responses and a median survival of only 37 weeks, the drug was considered inactive in this indication.

*Liposomal daunorubicin*. Liposomal daunorubicin (LD) was given to 13 patients at 120 mg m^−2^ every 3 weeks, but no responses were produced (Steele *et al*, 2001).

### Platinum compounds

*ZD0473*

A phase II trial (Giaccone *et al*, 2001) assessed the efficacy of the new generation platinum compound ZD0473 at 120 mg m^−2^ on day 1 of a 21-day cycle in mesothelioma patients that had relapsed after previous platinum-based chemotherapy. Two of 10 (20%) patients, experienced tumour shrinkage and five of 10 (50%) subjects had stable disease.

### Topoisomerase interactive agents

*Camptothecin analogues*

#### Irinotecan

The activity of single-agent irinotecan (125 mg m^−2^ given weekly for 4 weeks, every 6 weeks) in malignant mesothelioma was investigated by the CALGB (Kindler *et al*, 2000). In 28 patients evaluable for analysis, no complete or partial responses were observed and the median overall survival was 7.9 months, indicating that irinotecan, at least in this dose and schedule, had no antitumor activity and considerable toxicity (leucopenia, neutropenia, diarrhoea).

Furthermore, the combination of irinotecan and cisplatin was evaluated by a Japanese group (Nakano *et al*, 1999). A total of 15 chemonaive MPM patients were treated with irinotecan at 60 mgmg^−2^ on days 1, 8 and 15 and cisplatin at 60 mg m^−2^ on day 1, repeated every 28 days. A response rate of 26.7% (four partial responses) was observed and the median survival after chemotherapy was 28.3 weeks. In contrast to the trial conducted by the CALGB, Nakano *et al*,reported definite activity with only mild toxicity of this combination regimen and concluded that it warrants further clinical evaluation.

Recently, Verschraegen *et al*, (2001) published retrospective data of the same combination in 10 patients with peritoneal mesothelioma. Six courses of irinotecan 50 mg m^−2^ (day 1, 8 and 15) and cisplatin 50 mg m^−2^ (day 1) were administered every 4 weeks, either intraperitoneally or intravenously, and were well tolerated. The authors reported that 70% of the patients improved on treatment. Owing to the observed clinical benefit, a phase II trial is currently planned.

*Topotecan*. In order to evaluate the efficacy and toxicity of the camptothecin analogue topotecan in the treatment of MPM, the North Central Cancer Treatment Group treated 22 MPM patients with topotecan 1.5 mg m^−2^ daily for 5 days at 3-week intervals. 18 patients (86%) experienced grade 3 or 4 neutropenia. There were no objective responses seen and the median survival for all patients was 230 days (Maksymiuk *et al*, 1998).

### Antimicrotubule agents

*Vinca alcaloids*

*Vinorelbine*. Steele *et al*, (2000) administered vinorelbine to 64 patients with MPM at a weekly 30 mg m^−2^ dose for 6 weeks. A response rate of 21% (12 out of 64 patients) was observed and 63% of patients experienced disease stabilisation. Furthermore, the patients' quality of life was increased with respect to lung-related symptoms and physical well-being in general.

The same study group conducted a trial to evaluate the activity of vinorelbine at 30 mg m^−2^ on days 1 and 8 and oxaliplatin 130 mg m^−2^ on day 1 every 3 weeks in 21 MPM patients (Steele *et al*, 2000). Only two partial responses had been observed and toxicity had been significant. These inferior results could perhaps result from the large number of stage IV patients, the higher proportion of less favorable subtypes, and the inclusion of several participants showing a low performance status. At any rate, the addition of oxaliplatin to vinorelbine seems to bring no advantage, thus discouraging further studies in this doublet.

Recently, a British randomised phase III study has been initiated, comparing the efficacy of single-agent vinorelbine (30 mg m^−2^ up to 60 mg m^−2^) *vs* MPV (mitomycin 8 mg m^−2^, vinblastin 6 mg m^−2^, cisplatin 50 mg m^−2^) *vs* active best supportive care alone.

*Taxanes*

*Paclitaxel*. A total of 25 patients with MPM were given paclitaxel intravenously at a dose of 200 mg m^−2^ as a 3 h infusion every 3 weeks in a phase II study conducted by the EORTC Lung Cancer Cooperative Group (van Meerbeeck *et al*, 1996). No major objective responses were seen and the median survival time was only 39 weeks.

These disappointing results were confirmed by the CALGB, which administered paclitaxel at a slightly higher dose of 250 mg m^−2^, given as a 24-hour infusion every 3 weeks plus filgastrim (G-CSF) support to 35 patients with MPM (Vogelzang *et al*, 1999). Only three (9%) regressions of evaluable disease were observed and the median survival was 5 months.

Fizazi *et al*, (2000) conducted a phase II study of paclitaxel (200 mg m^−2^) and cisplatin (100 mg m^−2^), given to 18 patients on day 1 every 3 weeks. With only one partial response and a response rate of 6%, the paclitaxel–cisplatin doublet was considered ineffective and the trial was stopped early.

Bednar (1999) evaluated the combination of paclitaxel at 175 mg m^−2^ and carboplatin at an AUC of 5–6 in seven patients with malignant mesothelioma and reported more encouraging results. One patient with peritoneal malignant mesothelioma had a pathologically proven CR for a duration of 20 months. The overall median survival was 15 months from diagnosis and 12 months from the onset of treatment.

*Docetaxel*. The Eastern Cooperative Oncology Group assigned 19 patients with malignant mesothelioma to a phase II study of docetaxel 100 mg m^−2^, administered every 21 days. Only one patient achieved a partial response, with an overall response rate of 5%. The study group thus concluded that docetaxel was not effec-tive in the treatment of malignant mesothelioma (Belani *et al*, 1999).

Vorobiof *et al*, (2000), who also evaluated the activity of docetaxel using the same treatment schedule, reported only three partial remissions and six minor responses (25% or less reduction in tumour burden) in 22 MPM patients with median survival barely exceeding 12 months.

A clinical study further tested docetaxel at 60 mg m^−2^ in combination with irinotecan at 190 mg m^−2^ on day 1 of a 3-weekly cycle in 15 MPM patients (Knuuttila *et al*, 2000). No objective responses were achieved, and median survival was 8.5 months only. The study was finally discontinued owing to high toxicity and deficient activity levels.

### Antimetabolites

*Antifolates*

*Raltitrexed*. The EORTC has recently completed a phase II study of the quinazoline antifolate raltitrexed (ZD1694, Tomudex) at 3 mg m^−2^ as a single agent in the treatment of malignant mesothelioma, which is now in final analysis.

From 1999 to 2000, the Institut Gustave Roussy has treated 70 patients with a combination of raltitrexed (3 mg m^−2^) and oxaliplatin (130 mg m^−2^). Preliminary results show an encouraging response rate of 25% (14 of 57 patients evaluable for efficacy) and acceptable toxicity (Fizazi *et al*, 2000).

Based on these promising results, the EORTC initiated a randomised phase III study, evaluating the efficacy of the doublet raltitrexed (3 mg m^−2^)– cisplatin (80 mg m^−2^) *vs* cisplatin (80 mg m^−2^) alone.

*Pemetrexed*. A phase I study conducted in 1999 first investigated the activity of the multitargeted antifolate pemetrexed (LY231514, ALIMTA®) combined with cisplatin in patients presenting with solid tumours (Thodtmann *et al*, 1999). Of 11 mesothelioma patients evaluable for analysis, five experienced a partial remission (RR: 45%).

These promising data resulted in the largest phase III study ever conducted in patients with MPM, which was initiated in March 1999. The results of this single-blind trial were first presented at the ASCO annual meeting in May 2002. A total of 456 patients were randomised to receive either cisplatin–pemetrexed (cisplatin 75 mg m^−2^ and pemetrexed 500 mg m^−2^ on day 1, every 21 days) or cisplatin monotherapy in the control group. The combination of pemetrexed–cisplatin resulted in superior median overall survival, response rate, lung function and subjective quality-of-life measures (Vogelzang N http://www.asco.org/asco/ascoMainConstructor/1,47468,_12|002351,00.asp?cat=General+Oncology). The principal investigator concluded that based on these very promising data, pemetrexed–cisplatin should now be considered standard front–line therapy for patients with MPM.

Another phase I pemetrexed study demonstrated clinical activity in combination with carboplatin. A total of 27 patients were treated with various dose levels of pemetrexed and carboplatin on day 1 of a 21-day schedule (Hughes *et al*, 2002). In 25 evaluable patients, eight (32%) achieved a partial remmission and 14 experienced stable disease at various dose levels. Furthermore, a symptomatic improvement was documented in 19 cases.

A two-stage phase II trial has just been completed by Scagliotti and collaborators, investigating single-agent pemetrexed in the treatment of MPM. A total of 62 patients were given pemetrexed at 500 mg m^−2^ on day 1, every 3 weeks. Scagliotti *et al*, (2001) reported a response rate of 14.5% and a median survival of 10.7 months.

Furthermore, a randomised phase III trial comparing pemetrexed plus best supportive care *vs* best supportive care alone in previously treated patients with MPM is currently ongoing.

N*ucleoside Analogues*

*Gemcitabine*. The activity of single-agent gemcitabine at 1250 mg m^−2^ on days 1, 8 and 15 on a 28-day schedule was evaluated by the EORTC-Lung Cancer Group in 27 chemotherapy-naive subjects with MPM (van Meerbeeck *et al*, 1999). Two partial responses were achieved for an overall response rate of 7%. An additional 56% experienced disease stabilisation and overall median survival was 8 months.

The CALGB administered high-dose gemcitabine at 1500 mg m^−2^ on days 1, 8 and 15 in a 28-day cycle to 17 participants (Kindler *et al*, 2001). Only one minor regression and six cases of stable disease were reported in 13 assessable patients, with an overall median survival of 4.1 months. In contrast to these data, Bischoff *et al*, (1998) reported an encouraging response rate of 31% (5/16) with the same dose and schedule as the van Meerbeck study (gemcitabine 1250 mg m^−2^ on days 1, 8 and 15, every 28 days). Furthermore, seven additional patients reported a symptom relief via decreased pain or dyspnoea.

An Australian study group evaluated the doublet gemcitabine–cisplatin in 21 patients presenting with advanced MPM (Byrne *et al*, 1999). The subjects received gemcitabine at 1000 mg m^−2^ on day 1, 8 and 15 and cisplatin at 100 mg m^−2^ on day 1 of a 28-day cycle. This combination chemotherapy produced an encouraging response rate of 47.6% and an overall survival of 41 weeks. Furthermore, nine of 10 responding patients experienced significant relief of chest pain and dyspnoea. Subsequently, a multicentre study was initiated, evaluating the same chemotherapy regimen in 53 MPM patients (Novak *et al*, 2002). A response rate of 33% was achieved and the median survival time was 11.2 months. Response to treatment was accompanied by significantly improved global quality of life and respiratory function.

Contrary to the Australian trials, however, only four (15%) of 22 assessable subjects experienced a partial response within a European multicentre phase II study (Van Haarst *et al*, 2000) of gemcitabine at 1250 mg m^−2^ on days 1 and 8, in addition to cisplatin at 80 mg m^−2^ on day 1. The discrepancy between these studies may possibly result from the different treatment schedules, patient selection criteria and methodology applied in treatment evaluation.

In turn, Aversa *et al*, (1998) on evaluated the activity of the gemcitabine–carboplatin combination. A total of 20 patients were given gemcitabine at 1000 mg m^−2^ on days 1, 8 and 15 and carboplatin (AUC=5) on day 1, every 28 days for a median of 4.5 cycles. In 18 assessable subjects, a response rate of 16% and an 8.6-month median survival rate was achieved.

Recently, investigators at the University of Chicago have initiated a multicenter, randomised phase II trial of cisplatin–gemcitabine and the vascular endothelial growth factor (VEGF) inhibitor bevacizumab, which has shown preliminary evidence of activity in MPM.

## CONCLUSION

With rates of objective tumour regression ranging from 10 to 30% for cytotoxic monotherapy, diffuse pleural mesothelioma was considered to date to be widely chemoresistant. The most favourable responses to conventional chemotherapeutic agents were reported by the antimetabolites methotrexate and edatrexate.

In the past few years, a number of novel cytotoxic agents have been introduced into clinical oncology, the activity of which has also been tested in MPM therapy. Initial results produced by monotherapy based on new antimetabolites, along with platin combinations, provide encouragement. Preliminary results of the raltitrexed–oxaliplatin combination have shown promising activity. Single-agent gemcitabine and the drug combined with cisplatin appear to decrease symptoms associated with tumor load. In particular, pemetrexed with cisplatin has demonstrated superior median overall survival, response rate, lung function and quality-of-life measures in the largest randomised trial in MPM. These promising data suggest that finally effective chemotherapy exists for this aggressive disease and that pemetrexed–cisplatin will become a new systemic therapy standard.
